# Exploring intergenerational risk factors and mediators for child abuse potential in high-risk parents of young children

**DOI:** 10.3389/fped.2026.1750730

**Published:** 2026-05-18

**Authors:** Denise Keppler, Vanessa Reindl, Beate Herpertz-Dahlmann, Kerstin Konrad

**Affiliations:** 1 Child Neuropsychology Section, Department of Child and Adolescent Psychiatry, Uniklinik RWTH Aachen, Aachen, Germany; 2 JARA-Brain Institute II, Molecular Neuroscience and Neuroimaging, RWTH Aachen & Research Centre, Juelich, Germany; 3 Department of Child and Adolescent Psychiatry, Uniklinik RWTH Aachen, Aachen, Germany

**Keywords:** risk factors, child abuse potential, parental psychopathology, attachment, transgenerational transmission

## Abstract

**Background:**

Child maltreatment has profound adverse effects on various aspects of an individual's development and can even influence the next generation.

**Objective:**

This study investigated risk factors for child abuse potential within a population of highly burdened families and explored how these factors interact in the intergenerational transmission of maltreatment.

**Methods:**

Families receiving support from the German early prevention program “Frühe Hilfen” completed clinical interviews and standardized questionnaires at one time point. All subjects included met at least one risk factor for child abuse (inclusion criteria: low socioeconomic status, parental mental illness or teenage parenthood). Participants came from diverse cultural backgrounds. Child abuse potential was assessed using the CAPI, a screening instrument that measures parental stress and associated risk factors rather than actual abuse outcomes.

**Results:**

A multiple regression analysis identified parental psychopathology (b = .52, p = .024), attachment quality (b = 1.07, p = .001) and low socioeconomic state (b = −.83, p = .018) as key risk factors for child abuse potential. A mediation analysis indicated an indirect pathway whereby parental child maltreatment experiences were associated with poorer mental health (*b* = .32, *p* = .002), which in turn was linked to lower attachment quality (*b* = 1.71, *p* = .006) and higher child abuse potential (*b* = .12, *p* = .002). Affective disorders emerged as particularly significant risk factors among all mental disorders (b = 6.39, p = .015).

**Conclusion:**

These findings underscore the need for accessible psychiatric and psychotherapeutic support for parents with histories of childhood maltreatment to support healthy parent–child relationships.

**Clinical Trial registration:**

https://www.drks.de/drks_web/, German Clinical Trials Register DRKS00022075.

## Introduction

Child maltreatment^1^ (CM) or child abuse^1^ is internationally recognized as a serious public health problem. According to a recent meta-analysis, which included a total of 25 countries (from the following regions: North America, Europe, Developed Asia Pacific, East Asia, South Asia and America, West Asta and Africa), the prevalence of childhood exposure to physical violence is estimated between 16 and 17 percent (1). In 2023, the number of reports of child endangerment through neglect, psychological, physical, or sexual abuse reached a new high in Germany (73,700 cases reported), representing an increase of about 1,400 cases (+2%) compared to 2022 (with estimates suggesting a higher increase due to missing reports) (2). The COVID-19 pandemic has exacerbated the risk of child abuse due to lockdown measures, which have led to social isolation, unstable financial situations for families, and increased parental stress. Vulnerable families, in particular, have been disproportionately affected by these challenges (3).

CM often has profound negative long-term consequences, resulting in adverse physical, cognitive, psychological, and social outcomes for affected children (4). Especially chronic maltreatment (5) and exposure to various types of maltreatment have been found to contribute significantly to these negative outcomes (6). Early prevention programs are designed to support children and families to address risks and promote positive development before problems become severe. Despite existing prevention and intervention programs, their effectiveness remains limited. A meta-analysis indicated that early prevention programs yield only small and only short-term effects on parenting behavior, parental mental health and child's developmental outcome (7, 8). Given these challenges, the heterogeneity of current research findings and the variability in samples, methodologies, and measurement tools, there is a pressing need for further investigation into the risk factors and mechanisms underlying child maltreatment to develop more effective strategies for prevention and intervention (9, 10).

### Risk factors for child abuse

Most identified risk factors for CM have been found at the parental level (11). For instance, low family income (*r* = .166^2^) is a known factor for child abuse and neglect. There is also evidence that socially isolated parents (*r* = .037^1^) may be more prone to maltreat their children (12). Parenting stress is another significant risk factor for CM. A study by Crum and colleagues examined the relationship between parental stress, abuse potential, and children's social and behavioral outcomes, finding a consistent association between increased parental stress and higher abuse potential (13). Parents with mental health disorders (*r* = .259^1^) are potentially at an elevated risk of maltreating their children; for example, parents with depression are significantly more likely to have children for whom maltreatment was reported compared to parents without depressive symptoms (14, 15). Furthermore, parents who have experienced maltreatment during their own childhood [here referred to as early-life maltreatment; (ELM, *r* = .182^1^)] are at increased risk of maltreating their children, potentially contributing to the intergenerational transmission of abuse and neglect (16–18). A meta-analysis of 84 studies found that the risk of child maltreatment in families with parents who experienced ELM was nearly three times higher than in families without such histories. A recent cross-sectional study corroborates these findings by examining maltreatment transmission across three generations (grandparents, parents, and young adults), demonstrating continuity of maltreatment across all three generations with a medium effect size (19). Several theoretical mechanisms have been proposed to explain this transmission. From a *social learning* perspective (20), children who are exposed to harsh or abusive parenting may internalize these behaviors as normative strategies for discipline or conflict resolution, later reproducing them in adulthood. In parallel, the *stress sensitization* hypothesis suggests that early exposure to chronic or severe stress can dysregulate biological and psychological stress response systems, rendering individuals more reactive to later stressors such as parenting challenges or socioeconomic adversity (21, 22). This heightened reactivity may increase the risk of maladaptive coping or aggressive responses under pressure. However, contemporary empirical research indicates that while such mechanisms exist, they do not determine outcomes. Therefore, intergenerational models are best understood as *probabilistic* rather than deterministic frameworks (23).

There is a slight tendency in the literature showing that emotional maltreatment over physical or sexual abuse might be even more harmful in the intergenerational transmission of maltreatment (9, 24, 25). Whereby all types of maltreatment are considered to be a risk factor for the development of depression in adulthood, emotional maltreatment was additionally more strongly related to depression severity according to a larger meta-analysis (26). A recent systematic review by Younas and Gutman highlights several key risk factors at different levels. On a family level, the number of children in the family (*r* = .186), level of social support (*r* = .037) and partnership status (*r* = .285) emerged as most important risk factors. On a parental level low socio-economic status (SES, *r* = .166), young parental age (*r* = .140), substance abuse (*r* = .127), parental health issues (*r* = .259), parental stress (*r* = .184) and parental history of childhood maltreatment (*r* = .182) were identified as the most important risk factors (10). As these risk factors (level of social support, partnership status, SES, parental age, substance abuse, parental health issues, parental stress, parental history of child maltreatment) were most frequently associated with abuse potential in different studies, they were selected to be included in a joint analysis for the current study.

### Influence of interrelated risk factors

Research examining the interplay of various risk factors has shown that ELM experiences significantly increase the risk of clinically relevant depressive symptoms in parents (27–29). It is well established that ELM and depression often co-occur (30), suggesting that parental psychopathology may be a mediator in the intergenerational transmission of CM (24, 31). In addition to affective disorders, other psychiatric disorders associated with ELM experiences, such as borderline personality disorder (31, 32), substance abuse (33), post-traumatic stress disorder (34), and anxiety disorders (35, 36) could also increase the risk of child abuse. This link between parent's psychopathology and child abuse potential may be further mediated by the quality of the parenting behavior, difficulties in emotion regulation or parental competencies in reflective functioning (37, 38). Attachment, in particular, is often considered an additional mediator linked to parental mental health issues (39–43). Early in life, sensitive parents lay the foundation for a secure attachment by promptly and appropriately serving their infant's needs (44). However, children of parents with mental health issues, especially those exhibiting depressive symptoms, face an elevated risk of adverse developmental outcomes. A substantial body of evidence indicates that parents with depression are less responsive to their infants' cues, leading to poorer dyadic interactions and, consequently, weaker attachment quality (45). Attachment theory also suggests that parents who have ELM are at risk of developing poor attachment relationships, both with their parents and later with their own children (46). This is because their own caregiving experiences were marked by inconsistency and a lack of sensitivity, which led to the development of internal working models that view others as unreliable. These models then influence how these parents interact with their own children, impairing their ability to be emotionally present and apply positive discipline strategies during stressful or conflictual situations (47). An integrated perspective assumes that child abuse potential emerges from the interaction of parents’ early experiences, current psychological functioning, and parent–child relationship quality, rather than from isolated risk factors. This conceptual model directly informed the study hypotheses and guided our mediation analyses examining whether parental psychopathology and attachment quality statistically account for the association between parents' own experiences of childhood maltreatment and child abuse potential. Importantly, this model is probabilistic rather than causal and is tested here using cross-sectional mediation analyses to examine whether observed associations are consistent with the proposed theoretical ordering. Against this conceptual background, the present study further extends existing work by focusing on a highly burdened sample of families from Germany—a group that has received limited empirical attention despite the existence of broader international datasets. Only one other recent study examined multiple risk factors for CM within a German sample; however, this was a different sample from the one used in the present study. The focus here was primarily on early life maltreatment experiences among parents (necessary inclusion criterion) as a risk factor, which did not necessarily have to be present in this study. Moreover, while the previous study assessed the cumulative occurrence of risk factors, it did not explore their interrelations or underlying mechanisms (39). In the international context, there are more studies, and ours was able to successfully replicate some of the results. Notably, we employed structured clinical interviews to rigorously assess a wide spectrum of psychiatric disorders. To our knowledge, no prior study has integrated this breadth of clinically diagnosed mental health variables into a single theoretically-guided analysis of child abuse potential. This comprehensive approach adds a valuable perspective to the global literature by offering detailed insights from a local context with robust diagnostic precision.

In this study, we aimed to identify key factors associated with child abuse potential measured with the Child Abuse Potential Inventory (CAPI) which primarily reflects parental stress as a key indicator of child abuse potential (not abuse outcomes). We were particularly interested in interconnections between key risk factors, focusing on the intergenerational transmission of CM, within a high-risk German sample of parent-infant dyads, collected during and shortly after the Corona pandemic between 2020 and 2024. All participants were enrolled in the early prevention program (EPP) “Frühe Hilfen,” which offers low-threshold support to families in challenging circumstances with children up to 3 years (see “Materials and Methods” for more details) (41). Through a comprehensive assessment, including a full diagnostic interview for various psychiatric disorders and data on caregivers' own experiences of ELM, we conducted an in-depth characterization of the sample. First, we aimed to identify the most relevant risk factors which increase the risk for CM within our sample of highly burdened families (Research question 1). Based on the literature (10) and our integrated theoretical framework we hypothesized that the most significant risk factors for child abuse include parental risk factors (e.g., psychopathology, ELM experiences, socioeconomic status and attachment difficulties). We also expected to find interrelations between these main risk factors leading to our second research question, namely, to explore the relationships between the identified risk factors. A systematic review from 2022 identified parental depression, emotional availability of the caregiver, attachment and ELM in particular as possible mediators for harsh parenting style and problem behaviour within the offspring (47). Therefore, we specifically tested whether the association between parent's own ELM and child abuse potential was mediated by parental psychopathology and by quality of attachment to the child (How are the relevant risk factors related to each other?—Research question 2). We hypothesized that the association between parental risk factors and child abuse potential would be mediated by psychopathology and attachment quality. We tested whether different types of ELM and different mental health issues in parents affect the risk of child abuse more or less strongly (Research question 3). Regarding parental mental health, we hypothesized to find associations especially between the risk of child abuse and the presence of personality disorders, substance abuse, and affective disorders in the parent (17, 48, 49). We further anticipated that ELM is a risk factor regardless of the type of abuse, with a possible tendency for emotional maltreatment to emerge as a more severe risk factor, showing more interrelations with considered risk factors (25).

## Materials and methods

### Study design

Data were collected in Aachen and surroundings. Aachen is a city with about 260,000 inhabitants and is known by a large technical university https://www.aachen.de—Demografiemonitorings der Stadt Aachen (accessed on 21 January 2026). Ninety-three parent-child dyads were included in the present study. The study was part of a larger research consortium entitled “Understanding and Breaking the intergenerational cycle of abuse” (UBICA-II), funded by the Federal Ministry of Research and Education (BMBF). Parents of children aged ≤ 24 months or women in the third trimester of pregnancy who were supported by the early prevention program “Frühe Hilfen” were included in the study. Families who agreed to participate were visited at home to assess primary and secondary outcome measures at three measurement points. However, only data from the baseline survey (T0) was included in the analyses for this study. At T0, early intervention support has just begun (no more than 5 appointments).

The study was approved by the local Research Ethics Committee of the University Hospital RWTH Aachen (reference number EK 221/19) and was carried out in accordance with the Declaration of Helsinki. The study was registered at the German Clinical Trials Register (DRKS00022075 on 8 July 2020). For more information on the UBICA project see https://www.ubica.site/tp_fruehehilfen.html (accessed on 21 January 2026). The study protocol can be found under https://www.mdpi.com/2227-9067/11/3/267 (accessed on 21 January 2024). All participating parents provided informed consent for their own and their child's participation.

### Early prevention program (EPP)

The EPP service “Frühe Hilfen” is available for all families with children up to the age of three, from pregnancy onwards. They offer psychosocial support and home visiting services to families in need. Almost half of the families usually contact the EPP themselves. The other half is referred to the EPP via pediatricians, clinics, midwives and the youth welfare offices. The aim is to enable every child to develop healthily and grow up without violence (for more information in the EPP see https://www.fruehehilfen.de/grundlagen-und-fachthemen/grundlagen-der-fruehen-hilfen/was-sind-fruehe-hilfen/, accessed 26 January 2026). We included two EPP teams in Aachen and the surrounding areas with comparable team structures and professional backgrounds (i.e., family midwives, social workers, or pediatric nurses). Staff members had to provide informed consent to participate in the trial.

### Sample

We included all families (mothers or fathers, depending on who preferred to participate and their children) seeking support in the EPP who met the inclusion criteria (see Table 1) and were willing to participate. The risk factors were selected according to the descriptive data from clients in-need for longer term support of the EEP based on the annual reports (84).

**Table 1:** Inclusion criteria.

Caregiver	Child
- Must be supported by the EPP - Score >4 on at least one of the five subscales of the Adult Adolescent Parenting Inventory (AAPI version 2.1) - Or the presence of socioeconomic disadvantage (low socioeconomic status) - Or the presence of parental risk characteristics (mental illness) - Or teenage parenthood - And written informed consent provided by the caregiver	<24 months of age

EPP, early prevention program; AAPI, adult and adolescent parenting inventor.

### Measurements

#### Childhood experience of care and abuse questionnaire (CECA.Q)

Parental childhood maltreatment experiences were assessed at T0 using the German version of the CECA.Q, which is a retrospective self-report questionnaire on parent's behaviors towards children up to an age of 17 years. The German versions have been authorized by the author (A. Bifulco) of the original versions. The internal consistency of the CECA.Q in the German sample was.92 for Cronbach's *α* for both parents on the antipathy scale. For the neglect scale, Cronbach's *α* was .88 for the mother and .91 for the father (50). The questionnaire distinguishes between different types of maltreatment and assesses emotional maltreatment, physical and sexual abuse (51). Emotional maltreatment is assessed by 16 items related to parental care (8 items for antipathy and 8 for neglect) experienced by the mother and father, so that a total of four scales for emotional maltreatment can be formed. The items are rated on a 5-point Likert scale from “1 = yes definitely” to “5 = not at all” and can be added to a sum score (total score 16–80). There are defined cut-offs for each subscale. The presence of physical and sexual abuse is assessed by a “yes” or “no” question each. For the emotional maltreatment scale, Cronbach's alpha in the current sample was acceptable (*α* = .646). In order to compare the effects of mental vs. physical/sexual abuse, the four parental care scales of the CECA.Q (mother's and father's antipathy and neglect), were averaged and considered as a measure of emotional maltreatment in the current analyses [for a similar approach see Lehrl (52) and Hezhan et al. (53)]. The variable “presence of physical and/or sexual abuse” was dichotomized (1 = if physical and/or sexual abuse was experienced by the mother and/or father, or, in the case of sexual abuse, by others and 0 = if participants have not experienced physical or sexual abuse at all).

#### MINI DIPS

The MINI DIPS is a brief, structured diagnostic interview and offers a quick yet reliable diagnostic assessment of mental disorders according to DSM-5 and ICD-10 and was assessed at T0 only. The MINI DIPS Open Access provides an overview of the general burden of the persons examined and enables the documentation of important anamnestic information for practice and research. The DIPS interview has been regularly updated since the 1990s and its reliability, validity, and acceptance were repeatedly tested in large samples of outpatient, inpatient, and research populations and has demonstrated high interrater (*κ* ≈ .88–.98) and test-retest reliability (*κ* ≈ .80–.91) and good criterion validity (e.g., high agreement with SCID-5 and sensitivity/specificity >.85) (54). The interview contains dichotomous filter questions (yes/no) on various psychiatric disorders and distinguishes between current symptoms or past symptoms. If these filter questions are answered in the affirmative, these are followed by specific questions on the symptom criteria, predominantly in the “applies” and “does not apply” format. The ICD-10 or DSM-5 checklists can then be used to check whether the diagnostic criteria are met. If filter questions are answered in the negative, the specific questions are skipped, and the next disorder is addressed. Finally, a 9-point Likert scale is used to assess the level of function and the extent of impairment caused by the diagnosis(es) in question. In case of comorbid disorders, this item is answered only once considering the general level of function and extent of impairment across all diagnoses. The range from 0 to 3 is considered subclinical (slightly disruptive/not really). The range from 4 to 8 is considered clinically relevant (4: clearly disturbing, 6: strongly disturbing, 8: very strongly disturbing). In this paper, the functional level was included in the mediation analysis as a metric variable. In the present study, the internal consistency, as assessed by Cronbach's Alpha, was acceptable to good (*α* = .745). Interviews were conducted by an experienced psychologist which was supervised by a professor.

#### International personality disorder examination (IPDE)

The International Personality Disorder Examination/ICD-10 Module (German edition) is a semi-structured clinical interview to diagnose personality disorders as defined by the ICD-10 and DSM-IV classification systems. The interview is the most widely used screening tool for personality disorders based on worldwide field trials, which allows international application (55). It distinguishes between symptoms occurring before or after the age of 25. During implementation the clinician records the scores for each response on the IPDE Answer Sheet. After conducting the interview, the clinician can assign a definite, probable or negative diagnosis for each personality disorder. For this, precise scoring guidelines can be used. In the present study, only the areas of dissocial personality disorder and emotionally unstable personality disorder (borderline) were assessed. The agreement between different assessors is high. Kappa values are mostly between .65 and .72 for certain or probable diagnoses (56). The internal consistency, as assessed by Cronbach's Alpha, was acceptable for this study (*α* = .637).

#### Parenting stress inventory (PSI)

The PSI distinguishes between two main sources of parental stress: (1) child characteristics and behaviors that result in specific demands on parents and (2) limitations in parental functioning that impair the resources available to parents to cope with the demands of raising and caring for their child. The PSI contains 48 items corresponding to five subscales that record sources of stress that originate from the child's behavior and are associated with particular demands for the parents (distractibility/hyperactivity of the child, acceptability, demands, adaptability and mood) as well as seven subscales that record impairments of parental functions (attachment, social isolation, doubts about parental competence, depression, health, personal limitations, partner relationship). The PSI is a self-report (e.g., caregiver-report) questionnaire and can be used as a screening procedure to identify current threats to parent-child interaction at an early stage, including threats to the child's well-being due to high parental stress. T-norms are available for the main scales and for the overall scale of the PSI. Stanine norms are available for the subscales. The validity of the instrument has been examined in several studies that have reported substantial associations between PSI scores and various indicators of parental stress (57). In the present study, only the Stanine norms of the attachment subscale were included in the mediation analysis. Higher Stanine scores thereby indicate difficulties in bonding with the child. The attachment subscale measures a distant relationship with the child, which is expressed in an inability to empathize with the child and reliably assess his or her needs. A German version of the PSI showed high internal consistency reported in previous studies (*α* = .95) (57). Cronbach's alpha in our sample was good for the attachment subscale (*α* = .784). *T*-scores of the main parent scale were not included in the regression and mediation analysis because some of the measured constructs which are factored into the total score, e.g., depression, partner relationship and isolation, overlap with other variables in the models.

#### Child abuse potential inventory (CAPI)

The German version of the CAPI is a factor-analytically abbreviated and newly validated version of the Child Abuse Potential Inventory [CAPI, (58)]. It consists of 63 items and is a self-report (e.g., caregiver-report) screening tool for the detection of child abuse potential that is answered in a dichotomous agree/disagree format. Moreover, psychometric evaluation of the brief CAPI version (BCAPI) in German mothers and fathers supports its factorial validity (e.g., 59). The CAPI records current stress faced by parents as an indicator of possible risk to the child's well-being. This screening scale is primarily intended to identify and predict moderate to severe *physical child abuse and neglect* (It is not designed to assess the risk of sexual abuse or emotional neglect). The development of the CAPI was guided by the understanding that child abuse is influenced by a complex interplay of psychological and interpersonal factors. The scale includes the most predictive items to ensure it functions as a practical, reliable, and valid assessment tool for child abuse potential not abuse outcomes. The evaluation leads to a primary clinical scale for recording the parental stress level and three validity scales in order to record specific tendencies of response bias (social desirability, unreflected response behavior, inconsistent response behavior). In our sample, a total of 16 participants reached cut-off values on at least one of the three validity scales and therefore had to be excluded from the analyses. The raw value was converted into *T*-values (for risk samples) using norm tables and included in the current analyses. The internal consistency of the stress scale can be described as very good with a Cronbach's alpha of .91 (58). Cronbach's alpha for the current sample was good (*α* = .883) in the present sample.

#### Questionnaire for social support (FSozu)

The German F-Sozu self-report (e.g., caregiver report) questionnaire was conducted in the short form with 22 items. The F-Sozu operationalizes current social support as perceived or anticipated support from the social environment. The concept underlying the method is based on cognitive approaches and measures the subjective conviction of receiving support from others when needed as well as the assessment of being able to draw on resources from the social environment. The items are in statement form (e.g., “I have friends/relatives who are good listeners when I want to express myself”). The respondents indicate their level of agreement with these statements on a five-point Likert scale. Comparative norms for clinical and non-clinical groups are available for all questionnaire forms (60). Previous research has shown high internal consistency (*α* = .93) (60). Cronbach's alpha was acceptable to good for the social integration subscale (*α* = .693), which was used in the analysis.

#### Socioeconomic status (SES)

The socioeconomic status of the family was collected according to the GEDA study by the Robert Koch Institute (61). A total score representing the family SES is calculated by study stuff from the caregiver's highest educational qualification, the occupational status and the net household income. The scores range is between 3 and 21, whereby higher scores indicate a better SES.

### Statistical analysis

**Figure 1:**
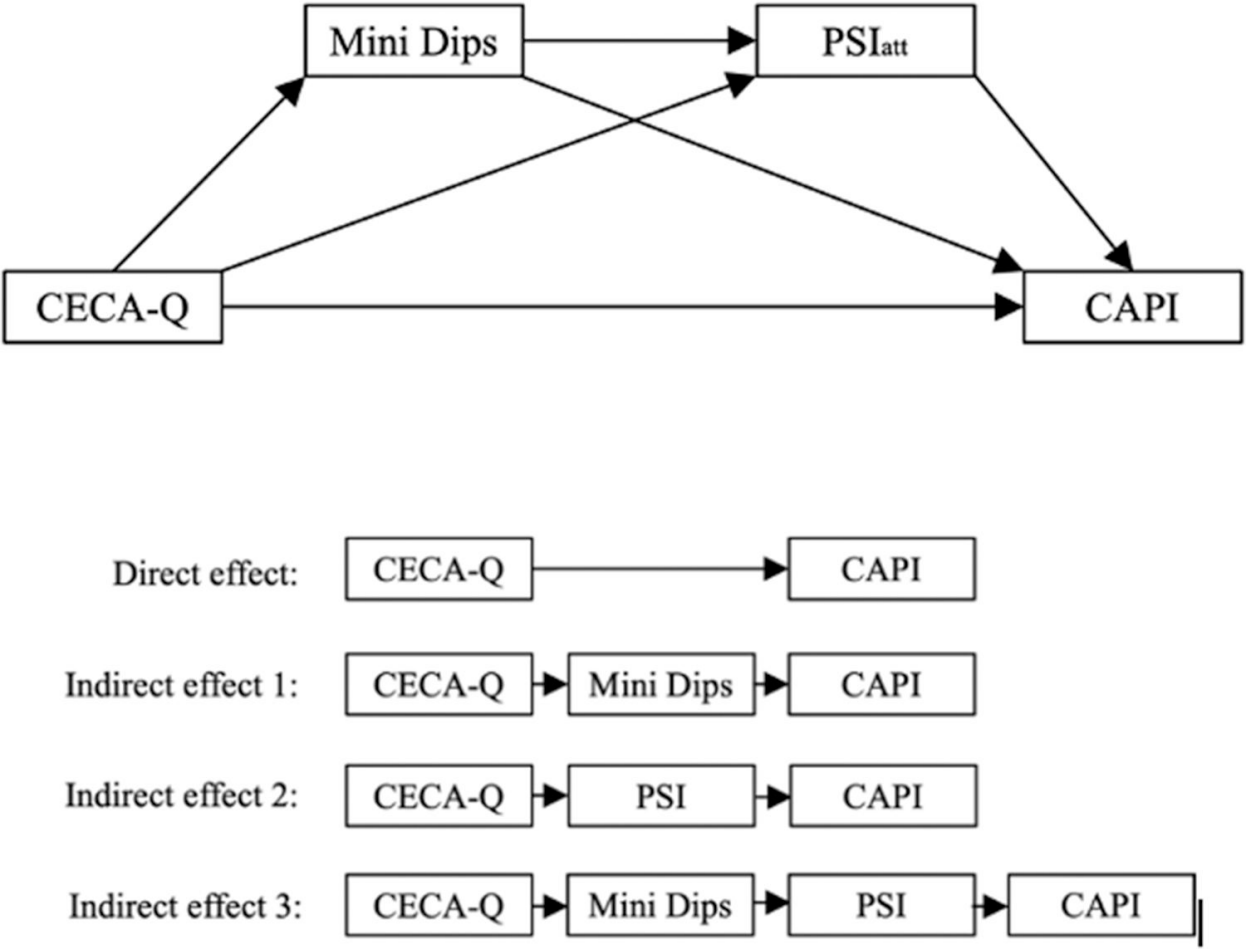
Mediation model with parental psychopathology and quality of attachment as mediators and visualization of direct and indirect pathways. CECA.Q, childhood experience of care and abuse questionnaire; PSI, parenting stress inventory; CAPI, child abuse potential inventory.

Data analyses were conducted in IBM SPSS Statistics 29 (IMB corp., Armonk, USA). All statistical tests were two-tailed and *p*-values <0.05 were considered statistically significant. First, we calculated descriptive statistics for the variables of interest (see Table 2). Second, we calculated Pearson correlation coefficients among the main variables (see Table 7). To explore our first research question, a linear regression analysis was run to assess the association between selected risk factors and child abuse potential, as the dependent variable, in our high-risk group. Selected risk factors were: parental psychopathology, parental childhood maltreatment experiences, family socioeconomic status, parenting stress (attachment), origin, level of social support, parental age and partnership status of caregiver. Risk factors were assessed at T0 and chosen based on a recent systematic review on parental risk factors on child maltreatment (10). Therein the above-mentioned risk factors have shown the most frequent significant associations with child abuse across 68 included studies (10). The *post-hoc* power analysis given *α* = 0.05, total sample size = 75, number of predictors = 7 and effect size *f*2 = 0.15 revealed a satisfactory power of 95% for this regression analysis using G-Power 3 (62). To address our second research question, a serial mediation analyses was run using the PROCESS macro (63) to explore whether the link between parental childhood maltreatment experiences and child abuse potential were mediated by caregivers psychopathology as well as by the quality of attachment. The independent variables were the parental emotional maltreatment scales of the CECA.Q as well as the physical and sexual abuse scales. Mediator variables were the MINI DIPS functional level (Mediator 1, M1) and the PSI attachment subscale (Mediator 2, M2). Dependent variable was the child abuse potential (CAPI) (see Figure 1). In total, two models were calculated, one for the CECA.Q emotional maltreatment scale and one for the CECA.Q physical/sexual abuse scale. We conducted two separate mediation analyses to specifically examine the effects of emotional maltreatment, which has been underrepresented in prior research (64). In contrast, parental physical and sexual abuse frequently co-occurred in our sample (55% co-occurrence), making it statistically and conceptually appropriate to analyze them together in a single model. Running separate models for these highly overlapping variables could have reduced statistical power and obscured shared effects. Indirect effects were tested by bias-corrected bootstrapping of the 95% confidence interval (*n* = 5,000 runs). Statistical power for detecting indirect effects was evaluated based on the simulation results reported by Fritz and MacKinnon (65). For medium-sized effects of both the *a* and *b* paths, a sample size of approximately *N* = 71 is required to achieve .80 power when using bias-corrected bootstrap confidence intervals. Given the present sample size (*N* = 73), the study was sufficiently powered to detect indirect effects of at least medium magnitude.

**Table 2:** Sociodemographic data.

*N* at T0	92
Sociodemographic characteristics	
Child's age in months (M, SD)	2.61 (3.65)
Caregiver's age in years (M, SD)	30.84 (6.94)
Sex of caregiver (f/m)	(85/7)
Sex of infant (f/m)	(50/42)
SES (M, SD)	8.54 (4.14)
Origin: EU including Switzerland (*n*/%)	57 (62.0%)
Turkey (*n*/%)	6 (6.6%)
Syria (*n*/%)	3 (3.3%)
Africa (*n*/%)	8 (8.8%)
Other (*n*/%)	17 (18.7%)
Number of siblings^a^ (M, SD)	0.84 (1.05)
Is the caregiver in a partnership?^b^ (yes/no)	(74/18)

SES, socioeconomic status.

^a^ Biological siblings, half-siblings and/or stepsiblings.

^b^ The partner did not necessarily have to be the biological father of the index child.

To explore our third research question, a linear regression analysis was conducted to assess which psychiatric disorders were associated with an increased potential for child abuse. The predictors included in the analyses were disorders that have been linked to heightened child abuse potential in previous studies (16, 19, 40, 66, 67). Therefore, affective disorders (depression), anxiety disorders, personality disorders, trauma disorders and substance abuse were included as binary predictors. The *post-hoc* power analysis given *α* = 0.05; Total sample size = 75, number of predictors = 5 and effect size *f*2 = 0.15 revealed a satisfactory power of 91% using G-Power 3 (62).

## Results

### Descriptive data on parental psychopathology and own experiences of childhood maltreatment

Main study analyses were run for either 75 (regression analyses) or 73 (mediation analysis) participants since 16 cases had to be excluded since the data in the CAPI could only be interpreted to a limited extent and furthermore only complete cases were included in the analyses, which led to the exclusion of 3 further participants for the regression analysis (*n* = 74) and of 4 further participants for the mediation analysis (*n* = 73). Missing data were minimal, with at least 90 of 93 participants completing each measure. Cases with missing values were retained and descriptive inspection revealed no indications of systematic demographic or risk differences between complete and incomplete cases.

Overall, 53% of all caregivers reported any kind of ELM. With respect to the PSI, 37% of caregivers reported clinically relevant attachment issues with their children and nearly 60% reported clinically elevated levels of parenting stress on the parental level. Four percent of participants had a CAPI T-score above 60 indicating an increased risk for child abuse. Descriptive statistics for the entire sample are presented in Tables 3–5.

**Table 3:** Caregiver characteristics.

*N*	92
Functional level MINI DIPS (M, SD)	3.24 (2.40)
Subclinical symptoms ≤3 (*n*/%)	48 (52.2%)
Clinical symptoms ≥4 (*n*/%)	44 (47.8%)
CECA.Q	
Emotional maltreatment (M, SD)	17.88 (7.17)
Mother antipathy ≥25 (*n*/%)	30 (32.3%)
Mother neglect ≥22 (*n*/%)	23 (24.7%)
Mother physical abuse (*n*/%)	25 (27.2%)
Father antipathy ≥25 (*n*/%)	19 (20.9%)
Father neglect ≥24 (*n*/%)	25 (27.5%)
Father physical abuse (*n*/%)	20 (21.7%)
Sexual abuse (*n*/%)	20 (21.7%)
PSI	
Attachment (M, SD)	9.83 (4.03)
Attachment SN ≥ 7 (*n*/%)	34 (37.0%)
Overall parental stress (M, SD)	75.45 (22.35)
*N*	75
CAPI (M, SD)	36.6 (10.11)
CAPI T ≥ 60 (*n*, %)	3 (4.00%)

CECA.Q, childhood experience of care and abuse questionnaire; PSI, parenting stress inventory; SN, stanine; CAPI, child abuse potential inventory.

**Table 4:** Prevalence of psychiatric disorders MINI DIPS and IPDE.

Mini Dips diagnoses	*N* = 93	%
Is there a disorder in the anxiety spectrum? (yes^a^/no)	(42/51)	(45.2/54.8)
Panic disorder? (yes^a^/no)	(18/24)	(19.4/25.8)
Agoraphobia? (yes^a^/no)	(8/34)	(8.6/36.6)
Social anxiety disorder? (yes^a^/no)	(12/30)	(13/32.3)
Specific phobia? (yes^a^/no)	(17/25)	(18.3/26.9)
Generalized anxiety disorder? (yes^a^/no)	(18/24)	(19.4/25.8)
Is there a disorder in the mood spectrum? (yes/no)	(42/51)	(45.2/54.8)
Manic episode? (yes^a^/no)	(1/41)	(1.1/44.1)
Major depression? (yes^a^/no)	(37/5)	(39.8/5.4)
Persistent depressive disorder? (yes^a^/no)	(4/38)	(4.30/40.86)
Is there an obsessive-compulsive spectrum? (yes^a^/no)	(11/82)	(11.8/88.2)
Is there a disorder in the trauma spectrum? (yes^a^/no)	(32/61)	(34.4/65.6)
Is there a disorder in the eating disorder spectrum? (yes^a^/no)	(7/86)	(7.5/92.5)
Is there a disorder in the addiction spectrum? (yes^a^/no)	(5/88)	(5.4/94.6)
Is there a disorder in the sleep disorder spectrum? (yes^a^/no)	(23/70)	(24.7/75.3)
Insomnia (yes^a^/no)	(20/3)	(21.5/3.2)
Hypersomnia? (yes^a^/no)	(3/20)	(3.2/21.5)
Is there an impulse control disorder (yes^a^/no)	(9/84)	(9.7/90.3)
Is there a psychosis (yes^a^/no)	(1/92)	(1.1/98.9)
Is there suicidal tendency? (yes^a^/no)	(27/66)	(29.1/71.0)
Is there a substance abuse disorder (yes^a^/no)	(5/88)	(5.4/94.6)
Is there a personality disorder? (yes/likely/no)	(13/11/69)	(14.0/11.8/74.2)
Impulsive type? (yes/likely/no)	(1/2/63)	(1.1/2.2/67.7)
Borderline type? (yes/likely/no)	(9/3/81)	(9.7/3.2/87.1)
Antisocial personality disorder? (yes/likely/no)	(3/6/84)	(3.2/6.5/90.3)

^a^ Symptom criteria may be present now or may have been met in the past.

**Table 5:** Overview of the prevalence of risk factors.

Risk factors	%
Psychopathology (MINI DIPS)	73
ELM (CECA.Q)	53
Attachment (PSI)	37
SES	45
Caregivers’ age	21
Partnership status	24
Origin	37

Psychopathology: at least one diagnoses; ELM: at least one type of ELM; attachment: stanine ≥7; SES: ≤7; Caregiver's age: ≤21 years; partnership status: caregivers without a partner; origin: families with migration background.

Forty-five percent of all caregivers suffered from a current or lifetime anxiety disorder and/or affective disorder. About 34% meet or met the diagnostic criteria for a posttraumatic stress disorder. About 30% reported a suicidal tendency at T0 or in the past. Approximately 26% probably or definitely fulfilled the symptom criteria for the presence of a personality disorder (either borderline or antisocial personality disorder). Only 5% reported a substance dependency disorder (see Table 4).

In our sample, twenty-two percent had two comorbid disorders, another 18% had three comorbid disorders and nearly 13% had four or five comorbid disorders (see Table 6). The most common comorbidities were anxiety and mood disorders with posttraumatic stress disorders.

**Table 6:** Prevalence of comorbid disorders.

Number of diagnoses	
*N*	*N* = 93
No diagnosis (*n*, %)	25 (26.90%)
One diagnosis	18 19.4%)
Two comorbid disorders	21 (22.6%)
Three comorbid disorders	17 (18.3%)
Four comorbid disorders	7 (7.5%)
Five comorbid disorders	5 (5.4%)

Correlations among study variables are shown in Table 7. Some variables showed only small deviations from normality. Therefore, Pearson's correlation coefficients were used to assess associations between variables, since it is robust to moderate non-normality and evaluates linear relationships between variables. For completeness, sensitivity analyses using Spearman's rank correlation were conducted and yielded similar results. The abuse potential (CAPI) correlated with caregiver's psychopathology (functional level from the MINI DIPS), the attachment subscale from the PSI and with SES. Additionally, some significant correlations with sociodemographic data were found., e.g., caregivers' age correlated with SES. Furthermore, the family's origin (coded with 1 = EU and Switzerland, 0 = any other origin) correlated significantly with caregiver's psychopathology as well as the level of perceived social support (see Table 7).

**Table 7:** Pearson correlations for main study variables.

Measurments*	CAPI	CECA.Q	SES	FL MINI DIPS	PSI_att_	FSozu	Origin	Partnership	Childs age	Childs gender	Caregivers age
CECA.Q	.20	1	−.14	.215	.30**	−.11	.05	.11	−.01	.12	−.07
SES	−.23*	−.14	1	.17	.13	.25*	.25*	−.03	.20	−.09	.47**
FL MINI Dips	.31**	.22	.17	1	.37**	.12	.47**	.13	.16	−.03	.07
PSI_att_	.41**	.30**	.13	.37**	1	.08	.07	−.02	−.01	.01	−.01
FSozu	.10	−.11	.25*	.12	.08	1	.28*	.06	−.03	−.04	.01
Origin	.14	.05	.25	.47**	.07	.28*	1	.17	.21	−.17	−.11
Partnership	.00	.12	−.03	.13	−.02	.06	.17	1	.08	.13	.08
CAPI	1	.20	−.23*	.31**	.41**	−.10	.14	.00	.03	−.01	−.06
Child`s age	−.03	−.01	.20	.16	−.01	−.03	.21	.08	1	.04	.06
Child`s gender	−.01	.12	−.09	−.03	.01	−.04	−.17	.13	.04	1	−.02
Caregivers age	−.06	−.07	.47**	.07	−.01	.01	−.11	.08	.06	−.02	1

CECA.Q, childhood experience of care and abuse questionnaire; SES, socioeconomic status; FL MINI DIPS, functional level MINI DIPS; PSI_att_, parenting stress index attachment subscale; FSozu, Fragebogen zur sozialen Unterstützung; CAPI, child abuse potential inventory.

*p < .05.

**p < .01.

Bold values indicate statistically significant results.

### Regression analysis I

In total we conducted two multiple regression analyses. In analysis I we explored which risk factors are associated with an increased risk for child abuse potential in our high-risk sample. Eight factors were tested simultaneously (see Table 8). Out of the 8 selected common risk factors taken into account only higher parental psychopathology (*β* = .235, *t*_(59)_ = 2.161, *p* = .023), lower family SES (*β* = −83, *t*_(59)_ = −3.147, *p* = .017) and lower quality of attachment (*β* = 1.07, *t*_(59)_ = 3.151, *p* < .001) emerged as significant factors for an increased child abuse potential. All other factors were not significantly associated with an increased child abuse potential. All other predictors were not significantly associated with an increased child abuse potential (see Table 8).

**Table 8:** Overview of all statistical results for regression analysis I.

Predictors	*B*	SE	*β*	t	*p*	95% CI
Psychopathology (FL MINI DIPS)	**.** **52**	**.** **457**	**.** **235**	**2** **.** **161**	**.** **024** *	**.51**; **2.33**
ELM (CECA.Q)	−.70	2.220	−.077	−.720	.732	−4.16; 4.68
Attachment (PSI)	**1** **.** **07**	**.** **278**	**.** **348**	**3** **.** **151**	**.** **001** **	**.32**; **1.43**
SES	**−.83**	**.** **287**	**−.364**	**−3** **.** **147**	**.** **018** *	**−1.47**; **−.33**
Caregivers’ age	.19	.160	.088	.805	.335	−.19;.45
Partnership status	−2.49	1.856	−.967	.067	.376	−.23; 1.28
Origin	3.28	.987	.245	.903	.235	−.18;.74
Social Support (FSozu)	−1.56	1.293	−.035	−.337	.280	−3.01; 2.14

CECA.Q, childhood experience of care and abuse questionnaire; CECA-Q was included in the regression analysis as a dichotomous variable (0 = no maltreatment was reported, 1 = at least one form of maltreatment was reported), CAPI, child abuse potential inventory, raw scores of attachment scale of the PSI, Parenting stress index (note: higher values indicate more attachment problems), SES, socioeconomic status, partnership status was included in the regression analysis as a dichotomous variable (0 = no partner at all, 1 = in a relationship, whereby the partner does not necessarily have to be the father of the child). Overall model significance *R*^2^ for regression analysis was .324.

* *p* < .05.

** *p* < .01.

Bold values indicate statistically significant results.

### Mediation analysis

**Figure 2:**
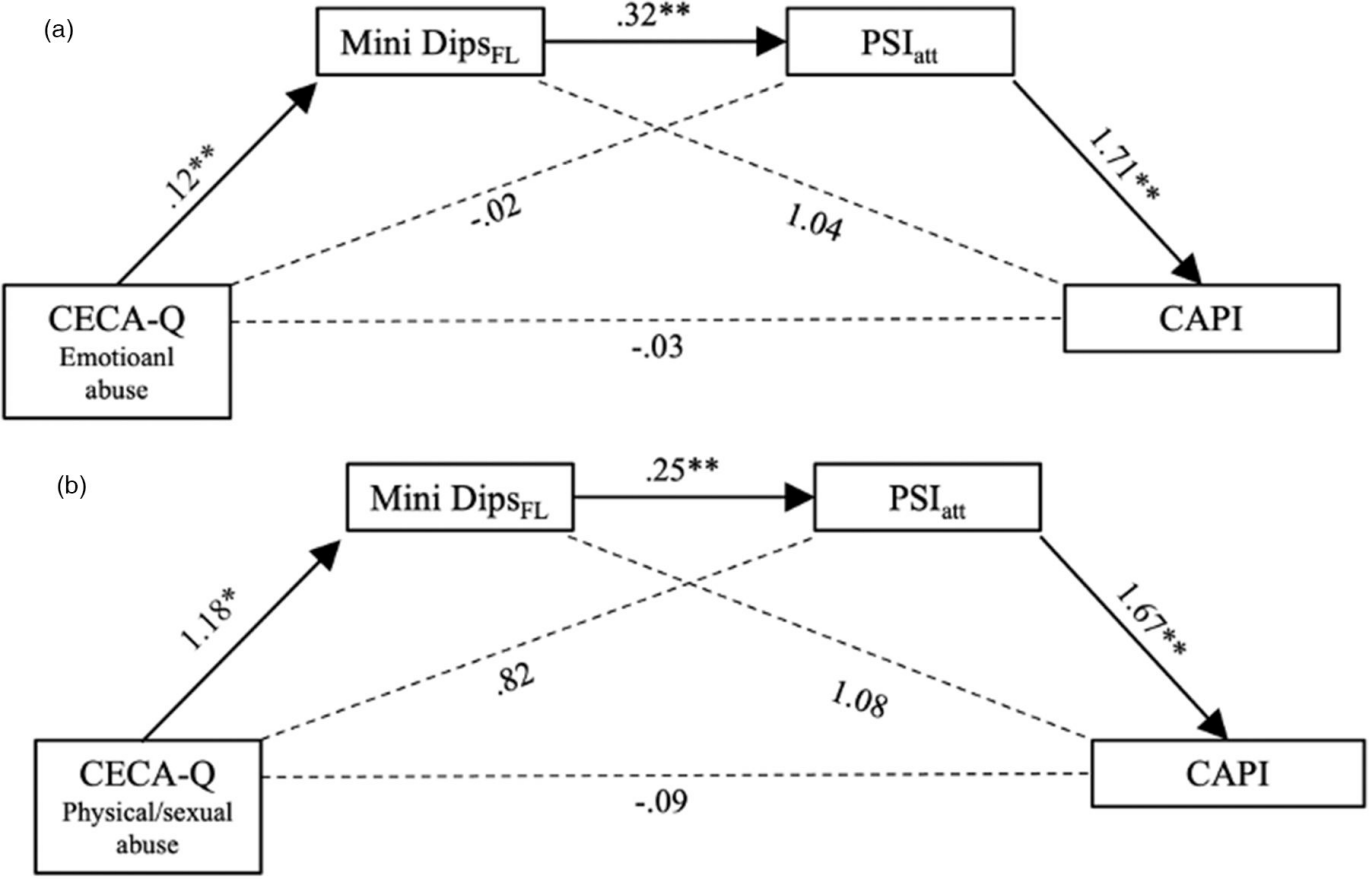
**(a)** Serial mediation model for the relationship between parental childhood maltreatment, namely parental emotional abuse, and the risk for child abuse. Mediators include parental psychopathology (mediator 1; MINI DIPS functional level) and quality of attachment (mediator 2; PSI). Unstandardized coefficients are depicted. *p < 0.05, **p < 0.01. Overall model significance *R*^2^ for model 1 was.28. **(b)** Serial mediation model for the relationship between parental childhood maltreatment, namely parental physical/sexual abuse and the risk for child abuse. Mediators include parental psychopathology (mediator 1; MINI DIPS functional level) and quality of attachment (mediator 2; PSI). Unstandardized coefficients are depicted. *p < 0.05, **p < 0.01. Overall model significance *R*^2^ for model 2 was.30.

We ran two mediation analyses to explore whether parental ELM experiences and the risk for child abuse potential were mediated by caregivers' functional level due to psychopathology and the quality of attachment to their child. SES was included as a covariate. The effects reported below are visualized in Figure 2a (Model 1 with emotioanl maltreatement) and Figure 2b (Model 2 with physical and/or sexual abuse). A visualization of direct and indirect mediation pathways can be found in Figure 1.

For Model 1, only indirect effect three (see Figure 2a) of emotional maltreatment on the child abuse potential (*b*_indirect3_ = .07; 95% CI [.009; .171]) was significant. Emotional maltreatment (*b* = .12; 95% CI [.046; .196], *p* = .002) and caregivers' psychopathology (*b* = .32; 95% CI [.125; .514], *p* = .002) and quality of child attachment (*b* = 1.71; 95% CI [.489; 2.931], *p* = .006) were significantly associated with current child abuse potential. All other mediation pathways were not significant (Table 9). In model 1 the total effect was not statistically significant. Nevertheless, this does not preclude meaningful indirect associations, particularly in multivariate models including SES and other covariates (68).

**Table 9:** Overview of all statistical results for mediations analysis.

CAPI scales	*b*	95% CI
Emotional abuse		
Total effect	.15	−.054; .432
Direct effect	−.02	−.339; .295
Indirect effect 1	.11	−.011; .302
Indirect effect 2	−.04	−.208; .113
Indirect effect 3	.**08**	**.011**; **.207**
Physical and/or sexual abuse		
Total effect	3.31	.761; 6.702
Direct effect	−.26	−4.728; 4.200
Indirect effect 1	1.19	−.048; 3.085
Indirect effect 2	1.55	−.102; 4.389
Indirect Effect 3	.**57**	**.006**; **1.648**

Indirect effect 1: relationship between parent's own ELM and child abuse potential mediated by parental psychopathology, indirect effect 2: relationship between parent's own ELM and child abuse potential mediated by quality of attachment, indirect effect 3: relationship between parent's own ELM and child abuse potential mediated by parental psychopathology and quality of attachment. Overall model significance *R*^2^ for regression analysis a was .319.

Bold values indicate statistically significant results.

For Model 2, analogously only indirect effect three (see Figure 2b) of physical and/or sexual abuse on the child abuse potential (*b*_indirect3_ = .49; 95% CI [.006; 1.420]) was significant again. Physical and/or sexual abuse (*b* = .1.18; 95% CI [.060; 2.294], *p* = .039) and caregivers' psychopathology (*b* = .25; 95% CI [.070; .431], *p* = .007) and quality of attachment (*b* = 1.67; 95% CI [.424; 2.919], *p* = .009) was significantly associated with current child abuse potential.

Both mediation analyses were also performed for the entire sample (without excluding subjects based on the CAPI validity scales). Including those did not change the results or conclusions (see Supplementary Table S1). Additionally, sensitivity analyses were conducted by re-ordering the mediators and outcomes; however, this did not result in significant alternative pathways.

### Regression analysis II

In the second multiple regression analysis we explored which psychiatric diagnoses (MINI DIPS) were associated with an increased child abuse potential, controlled for SES. All factors were tested simultaneously (see Table 10). Results revealed that only affective disorders (mainly Major Depression) were significantly associated with an increased risk for child abuse potential (*β* = 6.39, *t*_(69)_ = 2.792, *p* = .015). All other factors were not significantly associated with an increased child abuse potential (see Table 9). Although it should be noted that for substance abuse disorders, the number of subjects with a diagnosis was low (*n* = 5, 5.43%). On an explorative level, we also tested whether number of mental disorders was associated with an increased child abuse potential. We found a significantly positive association, indicating that a higher number of mental disorders (as dimensional variable) was associated with higher child abuse potential (*F*_(1,73)_ = 2.32, *p* = .003).

**Table 10:** Overview of all statistical results for regression analysis II.

Predictors	*B*	SE	*β*	*t*	*p*	95% CI
Anxiety disorder	−.30	2.166	−.045	−.436	.903	−5.25; 3.36
Affective disorder	**6** **.** **39**	**2** **.** **315**	**.** **306**	**2** **.** **792**	**.** **015** *	**1.86**; **11.07**
PTSD	.95	2.126	.128	1.339	.691	−1.38; 7.08
Substance abuse	4.02	5.082	.068	.682	.470	−6.64; 13.57
Personality disorder	6.71	2.842	.252	2.334	.115	−1.98; 12.28

PTSD, post traumatic stress disorder. Overall model significance *R*^2^ for regression analysis was .218.

* *p* < .05.

Bold values indicate statistically significant results.

## Discussion

The current study aimed to identify critical risk factors associated with child abuse potential in highly burdened families who are supported by a German early prevention program and explore how these factors interrelate.

Regarding *Research Question 1* (*Which risk factors increase the risk for CM within our sample of high-risk families?*), the results are in line with our hypotheses. Parental psychopathology, poor quality of attachment and a low SES were significant risk factors for an increased child abuse potential. Overall parental risk factors were more often significant ones, while socio-demographic aspects (origin, parental age, parental partnership status) were no direct risk factors for child abuse potential, which fits to the results in the literature (41). However, there were notable correlations between sociodemographic data and significant risk factors for child abuse potential. An association between parental age and SES was found, indicating that younger mothers have a lower socioeconomic status, and/or a low socioeconomic status may be a risk factor for early motherhood. This finding is in line with previous research showing a strong association between early motherhood, socio-economic disadvantage and low educational background (69). Adolescent mothers also report ELM experiences and mental health issues more often compared to adult mothers and are more likely to have an insecure attachment with their child (42). Additionally, origin correlated with psychopathology. Families from the European Union including Switzerland had a poorer functioning level due to psychopathology indicating a higher symptom burden. This seems unexpected at first, as families with an immigrant background may be more burdened, due to possible traumatic experiences in their home country or during the migration. Integration in the new home country may also be a burden. However, studies show that non-Hispanic whites compared to people of different ethnic groups are more likely to consider themselves mentally ill or in need of a mental health treatment. This is possibly due to the fact that mental illnesses as medical illnesses are understood differently across cultures (70). Furthermore, a systematic review from 2021 showed that ethnic minority groups often expressed greater public and/or self-stigma than White American groups regarding mental health problems (71). Accordingly, these groups may be less likely to report their psychiatric complaints. Origin also correlated with the extent of perceived social support, indicating less social integration and less social support for families with migration backgrounds. This seems not surprising since families with migration background must reintegrate themselves at first. Somewhat contrary to our expectations, ELM was also not a direct risk factor for child abuse potential. Previous evidence shows, however, that ELM has an indirect influence on child abuse potential via other mediators like parental psychopathology (24), which is in line with findings of our second research question.

Regarding *Research Question 2* (*How are the relevant risk factors related to each other?*) we hypothesized that parental psychopathology and quality of attachment play a potential mediating role in the intergenerational transmission of maltreatment. As expected, parental psychopathology and attachment mediated the relationship between ELM experiences in parents and their later child abuse potential. This aligns with prior research showing strong associations between ELM and various mental disorders (24, 37, 40, 66, 67, 72, 73), and the role of attachment as a key mechanism in this link has also been documented. For example, Wuebken et al. (39) examined a sample of 254 parents with a high prevalence of ELM experiences and found that the severity of parental ELM is associated with a higher child abuse potential, with attachment insecurity mediating this relationship. Further, a meta-analysis examining the impact of parental depression on attachment patterns showed that infants of depressed parents were less likely to show secure attachments and more likely to exhibit avoidant or disorganized attachment patterns. This is in line with extensive research evidence that depressed parents or parents with borderline personality disorder are less sensitive to their infants' cues, which typically results in a poorer quality of the dyadic interaction and poorer quality of attachment (31, 74, 75). However, it should be acknowledged that our mediation model does not imply a causal pathway. Parents and children may share genetic and environmental vulnerabilities for psychopathology, and children's behavioral challenges—shaped by these shared vulnerabilities—can increase parental stress and burden (76). Thus, while our findings are consistent with our tested mediation model, they may also reflect bidirectional or shared risk processes rather than a one-way causal relationship. Recognizing these dynamics is crucial, especially when interpreting findings for clinical implications. Further intervention and especially longitudinal studies are needed to prove such causal relationships. Psychiatric and/or psychotherapeutic treatment in parents might be helpful to reduce child abuse potential, especially if risk factors such as ELM or low SES are present (77).

Regarding Research Question 3 (*Can any specific parental psychiatric disorders or types of maltreatment be identified that particularly contribute to an increased risk for child abuse potential?*), to our knowledge, no study has simultaneously examined multiple psychiatric illnesses as risk factors for child abuse potential in one sample. In our second regression analysis, the most common psychiatric disorders (anxiety disorder, mood disorder, posttraumatic stress disorder, substance abuse disorder, borderline personality disorder or antisocial personality disorder) were included as potential risk factors for child abuse potential based on the literature. We found that in our sample, only parental depressive disorders represented a significant risk factor for increased child abuse potential. Although previous studies have already shown significant correlations with other psychiatric disorders, especially borderline personality disorder and substance abuse (31–33), the lower prevalence of these disorders in the current sample might have contributed to the lack of specific associations of these disorders to child abuse potential. Creating a hierarchy of mental health stigma, Hazell et al. found that mental health stigma varied in relation to psychiatric diagnosis. Personality disorders were much more stigmatized diagnosis than depression or anxiety (78). As a result, internalizing disorders may have been more likely to be reported in our sample. Note, however, that 16 cases were removed from the data analyses due to a lack of reliable responding in the CAPI. From these cases, 37.5% had a borderline or antisocial personality diagnosis which might have further biased our findings. Although no meta-analysis has yet examined various psychiatric disorders collectively, existing literature most frequently highlights associations between affective disorders, attachment, and child abuse potential (21). In our study ELM emerged as a risk factor for parental psychopathology regardless of maltreatment type. Most studies suggest that the effect of maltreatment type is indeed rather unspecific (24). In a meta-analysis psychological, corporal and sexual abuse were all significantly related to depressive disorders, however, emotional maltreatment was more strongly associated (25). Even if this study could not show that emotional maltreatment is more severe, emotional maltreatment and emotional neglect are less well-studied so that future studies should continue to investigate the effects of different types of maltreatment (79).

### Strengths and limitations

By addressing the impact of several risk factors at once in a sample of high-risk families, the current findings contribute to new insights regarding underlying mechanisms for the intergenerational cycle of maltreatment. This might help to develop interventions that address the most significant risk factors. Notably, the final models explained up to approximately one third of the variance in child abuse potential, indicating substantial explanatory power despite the relatively small number of predictors included. Moreover, our sample is a group that has been little studied to date with high prevalences of mental health issues and other risk factors and can be considered a representative sample for at risk and hard to reach clients. However, while this high-risk sample is well suited to the study's research questions, it also limits generalizability, as participating families may differ systematically from high-risk families who do not seek or receive preventive support. In addition, the exclusive inclusion of families already engaged in support services introduces potential selection bias. The cross-sectional design allows only limited causal interpretation. The relatively small sample size and consequent limited power may lead to difficulties to detect smaller effects or subgroup differences and the focus on families already in intervention may limits generalizability to high-risk families not receiving services. Detailed test diagnostics and clinical interviews for measuring parental psychopathology were conducted by experienced psychologists as objective diagnostic instruments. Some diagnostics however, relied on self-reports by the same caregiver which could reinforce some associations between study variables or underreporting Risk assessment is crucial in preventing child maltreatment since it can identify high-risk cases in need of child protection intervention. Empirical risk models for predicting child maltreatment are usually based on administrative data and statistically identified factors. Since such models often use factors that correlate with social disadvantage, there is a risk that they will reproduce systemic inequalities—for example, families with low incomes, multiple children, or previous convictions are disproportionately classified as “high risk.” This can lead to stigmatization: families are flagged, monitored more closely, and are more likely to have separation considered or initiated—even though the prediction is by no means reliable enough to justify exclusion or sanction decisions. In view of these risks, we want to emphasize that risk factors are not the same as causes and that a narrative, contextual assessment together with professional clinical judgment is necessary—rather than exclusively automated/empirical classification (80, 81). Only 4% of participants scored above the CAPI cut-off, which raises concerns about its predictive utility in this context. The CAPI may underestimate risk in families experiencing chronic adversity, such as high comorbidity and low SES, who may normalize high stress or underreport it due to stigma or social desirability bias. It remains unclear how the restricted range of CAPI scores affected analyses. Additionally, the CAPI was used as an outcome rather than actual abuse incidence. This underscores the importance of combining self-report tools with clinician judgment and collateral information in high-risk populations. Parental ELM was assessed using a retrospective measure which could lead to underreporting or recall bias (82), even though CECA.Q in general shows good psychometric criteria (83). Additionally, some psychiatric disorders occurred with high comorbidity rates, as did experienced types of abuse within subjects. This makes it difficult to truly disentangle the effects of individual disorders or specific types of maltreatment. However, the co-existence of mental disorders and multiple experienced forms of maltreatment are often the case, especially when investigating a high-risk sample. Although the PSI attachment subscale captures perceived difficulties in the parent–child relationship, it does not represent a comprehensive assessment of adult attachment representations as obtained with interview-based measures such as the Adult Attachment Interview or dimensional self-report instruments such as the ECR-R. Thus, future studies should replicate these findings using established attachment measures to further validate the mediating role of attachment processes. In addition, the results may be influenced by better powered analyses for mental illness of higher prevalence, especially mood disorders. Although, anxiety disorders were just as common as depression but were not significantly associated with child abuse potential.

### Implications for practice and further research

Our findings highlight the possible long-lasting impact of ELM on mental health issues and increased risk for CM to be transmitted into the next generation. ELM experiences cannot be completely reversed, but their effects later in life need to be addressed when becoming parents. Important implications are that highly burdened families with relevant risk factors (ELM, psychopathology and low SES) should have low-threshold access to outreach support services. These services should be equipped to recognize and address parental mental health issues, ideally within an integrated care model that includes attachment-focused interventions (such as parent-child psychotherapy and trauma-informed therapy for parents). Strengthening support in this way may help improve parent-child interactions and reduce maltreatment risk, since our results suggest that screening and treating parental mental health problems in high-risk families could be a key component of abuse prevention. Moreover, early intervention points—such as pediatric care, kindergartens, and family counseling centers—should routinely assess for these cumulative risk factors. Systematic screening could facilitate earlier, more tailored support. We recognize that our cross-sectional design limits causal interpretation, but the consistent associations observed across risk domains highlight clear targets for prevention and intervention strategies. Additionally, points of contact (e.g., pediatricians, kindergartens and other prevention services) should cover diagnostic assessments of certain risk factors by default. Future studies would benefit from taking several risk factors into account and considering the complex effects of the combination of different risk factors. Furthermore, it might be important for future studies to also consider protective factors that might buffer the effects of ELM on the next generations. To better understand the mechanisms driving the cycle of abuse, more detailed analyses of different types, severity and frequency of ELM may aid a deeper understanding of child maltreatment reoccurrence across generations. In addition, future studies should use a longitudinal study design to better identify causal relationships.

## Conclusion

The present findings suggest that parental depression and quality of attachment in particular play a mediating role in the intergenerational cycle of maltreatment. Parents with ELM experiences are at increased risk for mental health issues later in life accompanied by heightened parental stress and low quality of attachment with the child. Therefore, interventions that focus on ELM experiences and mental health issues in parents, in particular those with affective disorders might help to reduce the risk for child abuse potential and efficiently break the intergenerational cycle of maltreatment.

## Data Availability

The raw data supporting the conclusions of this article will be made available by the authors upon resonable request.

## Footnotes

^1^In the literature, the term *child maltreatment* is often used as an umbrella term encompassing abuse and neglect. In the present study, the terms *child maltreatment* and *child abuse* are used in a broad sense to refer to physical, emotional, and sexual abuse as well as neglect, consistent with the terminology used in the assessment instruments. When referring to previous studies, we aimed to retain the terminology used in the respective publications and to specify the type of childhood maltreatment investigated whenever possible.

^2^Reported effect sizes are based on a meta-analysis from 2018 by Mulder et al. (11).

## Supplementary material

Supplementary Table S1 Mediations analyses for the entire sample.
