# Supplementary material for: Exploring intergenerational risk factors and mediators for child abuse potential in high-risk parents of young children
Source: Front Pediatr. 2026 May 18;14:1750730. doi: 10.3389/fped.2026.1750730 (PMC13223124; doi:10.3389/fped.2026.1750730)
Supplement: Supplementary Table S1 — Mediations analyses for the entire sample. [file Table1.docx]

Table S1. Mediations analyses for the entire sample (Supplementary table)

| CAPI Scales | *b* | 95% CI |
| --- | --- | --- |
| Emotional Abuse | | |
| Total effect | .13 | -.175; .429 |
| Direct effect | -.07 | -.362; .429 |
| Indirect effect 1 | .15 | -.015; .223 |
| Indirect effect 2 | -.01 | -.208; .113 |
| **Indirect effect 3** | **.06** | **.006; .147** |
| Physical and/or Sexual Abuse | | |
| **Total effect** | **1.94** | **2.438; 6.702** |
| Direct effect | -.94 | -5.063; 3.175 |
| Indirect effect 1 | 1.28 | -.027; 3.167 |
| Indirect effect 2 | 1.21 | -.019; 3.291 |
| **Indirect effect 3** | **.39** | **.011; 1.206** |

*Note.* 1: Relationship between parent’s own ELM and child abuse potential mediated by parental psychopathology, Indirect effect 2: Relationship between parent’s own ELM and child abuse potential mediated by quality of attachment, Indirect effect 3: Relationship between parent’s own ELM and child abuse potential mediated by parental psychopathology and quality of attachment. Overall model significance R^2^ for regression analysis a was .281
